# Association between contrast-enhanced adenosine-stress perfusion cardiac magnetic resonance imaging and invasive coronary angiogram for the detection of coronary artery disease: A retrospective analysis

**DOI:** 10.12669/pjms.39.6.8008

**Published:** 2023

**Authors:** Sher Bahadar Khan, Rehmat Ghaffar, Muhammad Adil, Muneeb Ullah Jan

**Affiliations:** 1 Sher Bahadar Khan Associate Professor, Intervention Cardiologist, Lady Reading Hospital, Peshawar, Pakistan; 2 Rehmat Ghaffar Assistant Professor, Cardiac Imaging, MTI Hayatabad Medical Complex, Peshawar, Pakistan; 3 Muhammad Adil Assistant Professor, Cardiology, Lady Reading Hospital, Peshawar, Pakistan; 4 Muneeb Ullah Jan Resident Cardiologist, Department of Cardiology, Lady Reading Hospital, Peshawar, Pakistan

**Keywords:** CMR, Invasive coronary angiography, Coronary artery disease, Adenosine stress perfusion

## Abstract

**Objective:**

The objective of this study was to determine the diagnostic value of stress perfusion CMR for the detection of coronary artery disease.

**Methods:**

The was a retrospective cross sectional study in which 43 subjects were included from Cardiac MRI unit in the Hayatabad Medical Complex, Peshawar for study from 1^st^ April 2020 to 30^th^ November 2020. All the subjects who had been referred for stress perfusion CMR with suspected CAD were included in the study. Cardiac MRI both at rest and with adenosine stress perfusion was performed which was followed by invasive coronary angiography.

**Result:**

A total of 43 patients were enrolled for the detection or exclusion CAD who underwent stress perfusion CMRI and invasive coronary artery angiography. The study revealed strong and statistically significant association between positive stress perfusion CMR and positive coronary angiogram vs negative stress perfusion CMR and negative coronary angiogram (p= value 0.0001).

**Conclusions:**

Stress perfusion CMRI can be considered as a first line, relatively safe, noninvasive test with significant accuracy to diagnose coronary artery disease in patients with suspected CAD without subjecting these patients to invasive coronary angiogram

**Abbreviations:**
CMR: Cardiac magnetic resonance imaging,
CAD: Coronary artery disease,
FFR: Free Fractional Reserve,
MACE: Major Adverse Cardiovascular Events.

## INTRODUCTION

Cardiac Magnetic Resonance (CMR) is a sub-type of magnetic resonance imaging that focuses on understanding of coronary anatomy, regional cardiac function, and myocardial viability without needing intravenous contrast or ionizing radiation.^1^ CMR has found its utility in diagnosing coronary artery disease. The perfusion stress angiography a novel technique can also be used in the detecting of coronary artery disease. The most commonly used pharmacological agents for vasodilator stress are adenosine and dipyridamole due their excellent safety profile and high accuracy.^2^-^4^

The gold standard for determining the presence, location, and severity of coronary artery disease (CAD) is invasive coronary angiography even in this modern era with the latest gadgets.^5^,^6^ Invasive coronary angiography provides high spatial and temporal resolution for imaging of the coronary artery tree for catheter-based or surgical treatments, but it is an invasive and costly procedure and was linked to a small but significant incidence of morbidity (1.5%) and mortality (0.15%).^7^,^8^ As a result, for most patients with and at risk of coronary artery stenosis, a simple, non-invasive alternative technique for coronary angiography can bring considerable significant clinical and economic benefits.^9^ The field of noninvasive cardiac imaging is rapidly evolving. We aim to determine the association between stress perfusion CMR and invasive coronary angiogram and to determine the diagnostic value of stress perfusion CMR for the detection of CAD. The primary objective of the study was to determine the association between stress perfusion CMR and invasive coronary angiography and the diagnostic value of stress perfusion CMR for the detection of CAD.

## METHODS

This was a retrospective study in which 43 subjects were included from Cardiac MRI unit in the Institute Hayatabad Medical Complex, Peshawar. The ethical review board of Hayatabad medical complex approved the study reference number 1104; dated April 12, 2023.

### Ethical Approval

The study time considered from 1^st^ April 2020 to 30^th^ November 2020. All the subjects had been referred for stress perfusion CMR with stable ischemic heart disease and suspected CAD were included in the study.

###  Exclusion Criteria

The patients who had contraindications to CMR like atrial fibrillation, severe aortic valve stenosis, severe hypertension, intolerance to the pharmacological substances used in the study, the presence of acute coronary syndromes in the last 30 days and those with previous CABG were excluded. Both adenosine stress perfusion CMR and subsequent coronary angiography were performed. Myocardial perfusion measurements at rest and adenosine stress was performed in 43 patients (33 males 10 females, mean age 57.98 ± SD9.6years) at 1.5 Tesla MR Scanner with a Turbo Flash sequence. CMR data were reviewed by consultant cardiologist by visual assessment. Stress and rest perfusion was compared slice-by-slice on a picture archiving communication system. During contrast enhanced myocardial perfusion, perfusion defects were identified in terms of sub-endocardial decreased intensity. Adenosine infusion didn’t cause any untoward symptoms like shortness of breath or redness of face, similarly CMR didn’t have any side effects.

A segment of perfusion deficit on stress that was not seen at rest was classified as myocardial ischemia in at least three consecutive temporal images and at least two contiguous myocardial segments.^10^ A sub-endocardial or trans-mural delayed enhancement compatible with coronary distribution was classified as a myocardial infarction. Coronary angiography was performed and reported independently by two experienced intervention cardiologists in Institute Hayatabad Medical Complex, Peshawar without knowing CMRI report. Luminal narrowing was estimated visually and categorized as mild i.e., <50% narrowing of luminal diameter of the epicardial arteries, moderate i.e., 50% or more and severe i.e., 75% or more narrowing of the luminal diameter of the epicardial arteries. Stress-induced perfusion deficits were considered as positive stress perfusion CMR. These perfusion defects were correlated to angiographic stenosis, which include mild, moderate and severe stenosis based on visual criteria of coronary artery stenosis and categorized as positive coronary angiogram.

## RESULTS

### Statistical Analysis

Data was analyzed by using statistical SPSS software (version 16.0, SPSS). A total of 43 patients with suspected or known CAD underwent stress perfusion CMRI and invasive coronary artery angiography. The baseline demographics are summarized in Table-I. The majority of the patients were in the age group of 51-75 years old (79.1%) while 20.9% were in the age group of 30-50 years old. Ethnic variation included 23 Malay (53.5%), 01 Chitrali (2.3%), Hazarewal (44.2%). The mean age was 57.98 ± SD9.6; with a range of 30 to 72 (Fig.2).

**Table-I:** Baseline Demographics of Respondents.

Variable	Frequency, n(%)
***Age Group***	
30-50	9(20.9)
51-75	34(79.1)
***Ethnicity***	
Pashtun	23(53.5)
Chitrali	01(2.3)
Hazarwal	19(44.2)

**Fig.1:**
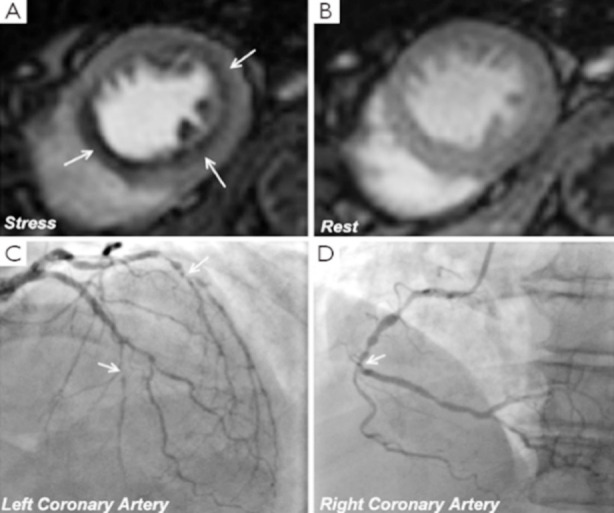
Perfusion CMR revealed stress-induced perfusion defects in all three coronary arteries (arrows A) and subsequent coronary angiogram showed three-vessel disease (arrows C and D) Adapted from Quant Imaging Med Surg by Ripley DP et al.^11^

Myocardial perfusion was measured at rest and during adenosine stress were performed in these patients. Perfusion defects were obtained and correlated to angiographic stenosis, which include mild, moderate and severe stenosis and categorized as positive coronary angiogram. The study revealed strong and statistically significant association between positive stress perfusion CMR and positive coronary angiogram vs negative stress perfusion CMR and negative coronary angiogram (p= value 0.0001), (Fig.1) (Table-II).

**Fig.2:**
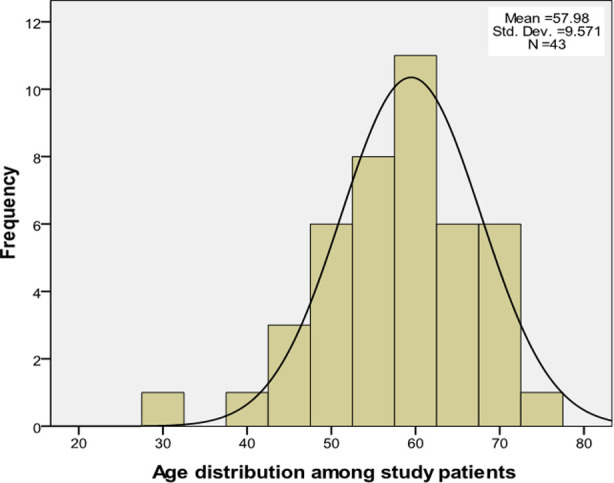
Age Distribution among study patients.

**Table-II:** Association between Stress Perfusion CMR and Coronary Angiogram.

Stress Perfusion CMR Result	Coronary Angiogram +ve	Coronary Angiogram -ve	Total
SP CMR +Ve	37	1	38
SP CMR -Ve	0	5	5
Total	37	6	43

(Chi-square test, p = 0.0001).

In our study the overall sensitivity for CMR depicting CAD with relevant stenosis was 97%, the specificity was 100% , the positive predictive value (PPV) was 100%, the negative predictive value (NPV) was 100% and the accuracy was 97.7%, (Table-III).

**Table-III:** Diagnostic value of stress perfusion CMR for the detection of CAD.

Sensitivity (%)	Specificity (%)	Positive predictive value (%)	Negative predictive value (%)	Accuracy (%)
97%	100%	100%	100%	97%

## DISCUSSION

Accurate assessment of myocardial ischemia caused by flow-limiting stenosis in the epicardial coronary arteries is important for the evaluation of chest pain and in the management of patients after coronary interventions. As myocardial blood flow at rest is not altered in stenosis up to 90% (due to autoregulation of the coronary circulation), vasodilator stress is needed to relax the arterioles and induces a noticeable difference between normal and ischemic myocardium. A recent meta-analysis concluded the superiority of perfusion myocardial perfusion scan over dobutamine stress echocardiography in diagnosis of coronary artery disease.^12^

Comparison between perfusion deficits at rest and during vasodilator stress can be used in ‘integrated’ imaging protocols to assess the clinical relevance of regional changes in the perfusion properties. Perfusion CMR has several advantages over nuclear medicine techniques and may be used as an alternative to these as cardiac MRI allows detailed evaluation of the coronary artery disease, left ventricular function, myocardial viability without the need of any radiation.^13^Areas of aberrant coronary artery blood flow under stress due to coronary artery stenosis or blockage were represented by myocardial ischemia, whereas areas of myocardial scar owing to previous infarction were represented by myocardial infarction. Cardiac MRI was found to be non-inferior to FFR (free fractional reserve) in patient with coronary artery disease.^14^-^15^ Both of these finding is thought to be the consequence of CAD. Our study result showed that high sensitivity, specificity, and accuracy of the combined stress-rest perfusion, and delayed enhancement were comparable to the earlier studies finding.^7^-^9^

In our study the overall sensitivity for CMR depicting CAD with relevant stenosis was 97%, specificity 100% , the positive predictive value was 100%, the negative predictive value was 100% and the accuracy was 97.7%. (Table-I) In addition to that we have also found that there is strong and statistically significant correlation between stress perfusion CMR and coronary angiogram (p= value 0.0001).The subjects who had positive CMR as the presence of stress perfusion defects were subjected to invasive coronary angiogram and noted to almost all patients had positive coronary angiogram which include mild, moderate and severe stenosis involving coronary arteries and similarly those subjects who had negative CMR noted to have a negative invasive coronary angiogram, (Table-IV).

**Table-IV:** Findings of Coronary Angiogram.

Coronary Angiogram	Number of Patients (%)
Normal Coronary arteries	06 (13%)
Mild coronary artery Steosis	10 (23%)
Moderate coronary artery Stenosis	14 (32%)
Severe coronary artery Stenosis	13 (30%)

The findings of our study strongly suggest that stress perfusion CMR can be used as a first line, relatively safe, noninvasive test with significant accuracy to diagnose coronary artery disease. In those subjects who have negative stress perfusion CMR can confidently be considered has normal coronary arteries without subjecting them to invasive coronary angiogram which is associated with a small but definite risk of morbidity (1.5%) and mortality (0.15%) . Those who have positive stress perfusion CMR can be considered for invasive coronary angiogram and if necessary for percutaneous coronary intervention depends upon severity of coronary artery stenosis. This study further intensifies the growing body of evidence regarding the utility of CMR for the diagnosis of coronary artery disease.

Recent studies have concluded that vasodilator stress induced is a predictor for MACE events even in asymptomatic individuals with coronary artery disease as well individuals with suspected of stable ischemic heart disease^16^,^17^ pointing to possible new uses of CMR. CMR might have a role in the detection of micro-vascular coronary artery disease even in the presence of obstructive coronary artery disease as reported by McGill et al.^18^, although in our study we didn’t see any such concomitant association.

### Limitations

It includes small sample size and single center study. Beside chest pain the other presenting symptoms of the patients and the complications encounter while doing CMR are not documented. In future such study should be conducted on large population keeping the co-morbidities and the complication develop under adenosine infusion. The lack of local data also makes it difficult to compare our findings with other local data.

## CONCLUSION

CMR can be considered as a first line, relatively safe, noninvasive test with noteworthy accuracy to diagnose coronary artery disease in patients with suspected CAD without subjecting these patients to invasive coronary angiogram which is associated with a small but definite risk of morbidity (1.5%) and mortality (0.15%). We demonstrated routine applicability, feasibility and safety protocol in outpatients of CMR.

### Authors’ contributions:

**SBK:** Concepts, Clinical studies, Data acquisition.

**RG:** Design, Data analysis.

**SBK** & **RG:** Literature search.

**SBK**, **RG** & **MJ:** Manuscript preparation.

**MA** & **MJ:** Manuscript editing.

**MJ:** Manuscript review.

**SBK:** Responsible for the integrity of the work.

## References

[1] Whittington B, Dweck MR, van Beek EJ, Newby D, Williams MC (2023). PET-MRI of Coronary Artery Disease. J Magn Reson Imaging.

[2] Garcia-Baizan A, Millor M, Bartolome P, Ezponda A, Pueyo JC, Gavira JJ (2019). Adenosine triphosphate (ATP) and adenosine cause similar vasodilator effect in patients undergoing stress perfusion cardiac magnetic resonance imaging. Int J Card Imaging.

[3] Kong H, An J, Cao J, Tang Z, Tian J, Yong J (2023). Adenosine triphosphate (ATP):a safe and effective vasodilator for stress perfusion cardiac magnetic resonance imaging. Clin Radiol.

[4] Pezel T, Garot P, Hovasse T, Unterseeh T, Champagne S, Kinnel M (2021). Vasodilatation stress cardiovascular magnetic resonance imaging:Feasibility, workflow and safety in a large prospective registry of more than 35,000 patients. Arch Cardiovasc Dis.

[5] Bajracharya P, Acharya KP, Banerjee SK, Ahmed CM, Alam MM, Jahanara AR, Sheikh N (2020). Correlation between myocardial Strain by 2-D speckle-tracking echocardiography and angiographic findings by coronary angiogram in stable angina. Maedica.

[6] Storey P (2022). Cardiac imaging:2022 update. Aus J Gen Pract.

[7] Goerne H, De la Fuente D, Cabrera M, Chaturvedi A, Vargas D, Young PM (2021). Features of Complications after Coronary Interventions and Surgical Procedures. Radiographics.

[8] Murgia A, Balestrieri A, Crivelli P, Suri JS, Conti M, Cademartiri F (2020). Cardiac computed tomography radiomics:An emerging tool for the non-invasive assessment of coronary atherosclerosis. Cardiovasc Diagn Ther.

[9] Feldman DI, Latina J, Lovell J, Blumenthal RS, Arbab-Zadeh A (2022). Coronary computed tomography angiography in patients with stable coronary artery disease. Trends Cardiovasc Med.

[10] Cury RC, Cattani CA, Gabure LA (2006). Diagnostic performance of stress perfusion and delayed enhancement MR imaging in patients with coronary artery disease. Radiology.

[11] Ripley DP, Motwani M, Plein S, Greenwood JP (2014). Established and emerging cardiovascular magnetic resonance techniques for the assessment of stable coronary heart disease and acute coronary syndromes. Quant Imaging Med Surg.

[12] Haberkorn SM, Haberkorn SI, Bonner F, Kelm M, Hopkin G, Petersen SE (2021). Vasodilator myocardial perfusion cardiac magnetic resonance imaging is superior to dobutamine stress echocardiography in the detection of relevant coronary artery stenosis:a systematic review and meta-analysis on their diagnostic accuracy. Front Cardiovasc Med.

[13] Jung JH, Yoon YE (2017). Advanced noninvasive cardiac imaging using cardiac magnetic resonance imaging in the diagnosis and evaluation of coronary artery disease. Ann Nucl Cardiol.

[14] Nagel E, Greenwood JP, McCann GP, Bettencourt N, Shah AM, Hussain ST (2019). Magnetic resonance perfusion or fractional flow reserve in coronary disease. N Engl J Med.

[15] Lee VS, Resnick D, Tiu SS (2004). MR imaging evaluation of myocardial viability in the setting of equivocal SPECT results with (99m)Tc sestamibi. Radiology.

[16] Pezel T, Garot P, Kinnel M, Unterseeh T, Hovasse T, Champagne S (2021). Prognostic value of stress cardiovascular magnetic resonance in asymptomatic patients without known coronary artery disease. Eur Radiol.

[17] Ng MY, Chin CY, Yap PM, Wan EYF, Hai JSH, Cheung S (2021). Prognostic value of perfusion cardiovascular magnetic resonance with adenosine triphosphate stress in stable coronary artery disease. J Cardiovasc Magn Reson.

[18] McGill L, Gill C, Schoepf UJ, Bayer RR, Suranyi P, Varga-Szemes A (2022). Visualization of Concurrent Epicardial and Microvascular Coronary Artery Disease in a Patient with Systemic Lupus Erythematosus by Magnetic Resonance Imaging. Top Magn Reson Imaging.

